# Surfboard riders are at risk of low energy availability – A pilot study

**DOI:** 10.1177/02601060231204927

**Published:** 2023-09-29

**Authors:** Mackenzie Baker, Pamela Magee, Josh Williamson

**Affiliations:** 1 School of Sport, 2596 Ulster University, Coleraine, UK

**Keywords:** Surfing, low energy availability, relative energy deficiency, sport, dietary analysis

## Abstract

**Background:**

Surfing is a rapidly growing sport and recreational activity. The previously reported, intermittent high-intensity energetics of surfing place athletes and recreational participants at risk of low energy availability (LEA).

**Aim:**

As such, this pioneering study aims to be the first to investigate LEA risk and the second to investigate dietary intake in surfers.

**Methods:**

Twenty-one intermediate and advanced surfers (female – 5, male – 16) were recruited to complete an online self-administered questionnaire and 4 consecutive 24-hour food logs to establish LEA risk and asses dietary intake. The Low Energy Availability in Female Questionnaire and Eating Disorder Examination Questionnaire were used to identify at-risk individuals in females and males, respectively, with respective cut-off's of ≥8 and ≥2.3.

**Results:**

Fifty-seven percent were classed as at-risk of LEA (50% and 80% in males and females, respectively). No significant relationship of competitive status, surfing ability and body mass index on risk classification was found. However, a non-significant medium effect of age was observed (p = 0.338, R = 0.549). And 77% of the 70 total analysed food records showed inadequate carbohydrate (CHO) consumption.

**Conclusion:**

In summary, an alarmingly high portion of surfers are at risk of LEA and dietary inadequacy. Future studies should confirm whether surfing organisations need to intervene, by addressing limitations of the present study including a small sample, which was heavily biased away from female and high-level competitors.

## Introduction

From an estimated 37 million surfboard riders (surfers) in 2016 (Klick et al., 2016) the popularity of surfboard riding (surfing) has risen exponentially alongside its inaugural inclusion in the Tokyo 2021 Olympic Games (International Olympic Committee, 2021). And 22% more people viewed the World Surfing League finals online, in 2022 compared to 2021 (8.3 million viewers) (World Surf League, 2022) and surfing equipment's market share was predicted to grow by 5.11% between 2022 and 2027 (IMarc Group 2022). Surfing is cementing itself as a popular mainstream recreational activity, competitive sport and global industry. As such, ancillary factors including nutrition grow in interest as a means of influencing performance, and health in the surfing population, yet nutrition-related literature in surfing is scant.

Whilst physical demands are heavily influenced by environmental factors (Minghelli et al., 2019), athletes compete in up to 5 × 20–300-minute heats (Association of Surfing Professionals, 2021), totalling 100-minutes of aerobically dominant activity with frequent high-intensity anaerobic efforts and short recovery periods (Farley et al., 2017) in a single competition day. Estimated energy expenditure of ∼500 calories per hour (Barlow et al., 2014; Meir et al., 1991) and observed mean heart rate (HR) of 64–85% HR maximum (Barlow et al., 2014) corroborate the strenuous demands of surfing. Despite HR being greater in competition (Farley et al., 2017), recreational surfing is typically of much greater duration (∼4–5 hours) (Méndez-Villanueva et al., 2005) suggesting that both competitive and recreational surfers are mutually exposed to strenuous physical demands similar to that of swimming, tennis, cycling (Felder et al., 1998), Australian Rules Football (AFL), and soccer (football) (Farley et al., 2017).

Low energy availability (LEA) (<30kcal/kg of fat-free mass (FFM)) describes the failure to meet energy requirements to support all human physiological functions independent of exercise (Areta et al., 2021). This energy mismatch occurs for a variety of reasons including, but not limited to practical barriers associated with meeting erroneously high daily energy requirements, and/or disordered eating/eating disorders (DE/ED) (Mountjoy et al., 2018). The symptomology of LEA has been characterised as Relative Energy Deficiency Sport (RED-S) and is prevalent among athletes (Melin et al., 2019). Indeed, recent findings in female soccer athletes (67% according to Magee et al., 2020, 64.7%, Luszczki et al., 2021), and female AFL athletes (30%, Condo et al., 2019), indicate a high prevalence of LEA/RED-S risk. It should also be noted that male athletes are at risk of LEA as indicated by McGuire et al. (2022) who found 70% of elite male Gaelic footballers to be in a state of LEA, and Lane et al. (2021) who found a mean energy availability (EA) of 28kcal/kg FFM in recreationally trained male endurance athletes.

Despite the aforementioned data showing an alarming theme of LEA in athletic populations from sports with similar physiological demands to surfing, practical and accuracy concerns associated with direct LEA assessments and screening tools, heavily limit the validity of findings (Burke et al., 2018; Loucks et al., 2011; Sim and Burns, 2021). This is especially controversial in male samples owing to a lack of validated male LEA screening questionnaires, and consensus of male LEA thresholds (Lane et al., 2021).

Despite surfing's growing popularity, EA is yet to be investigated in surfers, and to date, only one study (Felder et al., 1998) has investigated dietary intake, nutrition knowledge and energy expenditure in surfers. Although there were many methodological limitations (i.e. small sample size, self-reported food diary and the use of energy expenditure estimations derived from aquatic sports), all 10 elite female competitive surfers failed to achieve energy balance, and nutrition knowledge was found to be poor. Further, the paucity of food availability and food safety concerns during competitions in remote coastal locations creates nutrition challenges unique to surfers (Felder et al., 1998) that may amplify inadequacy.

The primary aim of the present study was to investigate whether surfers are at risk of LEA. Secondary aims included assessing the dietary intake of surfers and comparing them to sports nutrition and Australian Nutrient Reference recommendations. Finally, identify whether competitive status and/or surfing ability correlates with LEA risk. We hypothesised that a high prevalence of LEA risk would be observed, particularly among competitive surfers, compared to recreational surfers, and the carbohydrate intake of competitive surfers would fall significantly below recommendations.

## Methods

### Study design

After approval was granted by the Ulster University Research Ethics Filter Committee (FCBMS-22-144), 21 surfers (male − 16, female – 5) were recruited from Australia and New Zealand using email and social media to complete an online self-administered questionnaire and 4 consecutive 24-hour food log hosted on Google Drive (Google LLC, California, USA). Contact was accompanied by study overview, and instructional videos recorded and hosted using Loom (San Francisco, USA), a 30-second promotional video, sharable flyer, and a hyperlink to a separate webpage (Linktree, Darlinghurst, Australia) providing interested individuals access to detailed study information and required questionnaires to participate. Questionnaires commenced by obtaining informed consent from participants. Participants were incentivised with a free 1-year Stab Premium surf media subscription (Stab Magazine, Byron Bay, Australia). Given the novelty of research in surfing, power calculations were not used to determine sample size targets. Inclusion criteria are detailed below.

### Inclusion criteria

- ≥18 years of age
- Otherwise, healthy, free from underlying medical conditions that may affect dietary intake (chronic disease, namely type 1 diabetes)
- Have consistently participated in surfing ≥2 times per week, for the prior 3 months
- Have been a surfer for ≥2 years prior to participation
- Self-identify as intermediate or above level of surfing ability (at least able to ride inside the tube, perform cutbacks and re-entries)

### Exclusion criteria

- <18 years of age
- Current use of medication that may affect energy balance homeostasis or dietary intake
- Unable to complete the studies requirements
- Currently injured and unable to exercise /surf to a level considered >8/10 of their best ability
- Consistently participate in surfing <2 times per week in the past 3 months
- Has been a surfer <2 years

Self-report as a beginner surfer, or < intermediate ability.

Dietary intake was assessed by online self-reported 4-day consecutive food log with written and screen-recorded instructions, and portion size reference sheet to enhance detail and attenuate misreporting (Vargas-Alvarez et al., 2021). Participants were encouraged to create a photo record of food and drink consumed as a resource for later referral, when completing food records. Participants were informed that completing all food logs had to be done in real time and retrospective completion was not permitted. Participants were prompted to confirm whether the data entered applied to the same day of recording with a yes/no response. No responses were excluded from analysis. Food logs were manually entered into FoodWorks v.10.0 nutrition analysis software by one individual researcher to reduce variation.

LEA risk for female participants was assessed using the validated 25-item Low Energy Availability in Female Questionnaire (LEAF-Q) through self-reported symptoms including gastrointestinal symptoms, injury frequency, and menstrual dysfunction, a cut-off of ≥8 was used to identify at-risk individuals (Melin et al. 2014). For male participants, LEA risk was propounded using the Eating Disorder Examination Questionnaire (EDE-Q). A total score cut-off of ≥2.3 (Torstveit et al., 2019) or ≥1 for eating pathology or ≥3 dietary restraint subscales (Brook et al., 2019) was used to classify risk.

### Statistical analysis

Statistical Packages for Social Sciences (SPSS, version 27) was used for statistical analysis. EDE-Q or LEAF-Q cut-off in male and female participants, respectively, indicated risk classification and expressed as a percentage of total sample. All data was assessed for normality using the Shapiro–Wilk test of normality. For normally distributed data, mean ± standard deviation (SD) was reported. Median and interquartile range (IQR) was reported for variables that were not normally distributed. Independent samples t-test and Mann–Whitney U test were used to identify significant differences between LEA questionnaire score in normally and not normally distributed data, respectively. Chi-square was used to compare differences for activity characteristics and LEA risk classification. A p value of 0.05 was applied for significance.

## Results

Age, biometrics and LEA classification of eligible participants are summarised in Table 1. Of the 31 participants, 21 were eligible for inclusion, of which 57% (males – 50%, females – 80%) were classified as being at risk of LEA. Differences in continuous variables between LEA risk classification are summarised in Table 2.

**Table 1.** Participant characteristics.

	All participants n = 21	Male n = 16	Female n = 5
Age (years)	30 (28 – 40.5)*	35.25 ± 7.83	29.80 ± 2.63
Weight (kg)	77.76 ± 10.86	81.25 ± 8.53	64 (59.5 – 75)*
Height (m)	1.77 ± 0.08	1.80 ± 0.06	1.67 (1.65 – 1.73)*
BMI (kg/m^2^)	24.62 ± 1.72	25.02 ± 1.40	23.37 ± 2.20
At risk of LEA^1^	n = 12 (57%)	n = 8 (50%)	n = 4 (80%)
Not at risk of LEA	n = 9 (43%)	n = 8 (50%)	n = 1 (20%)

Data presented as mean ± SD unless specified otherwise.

*Value presented is median (IQR) due to data not being normally distributed as determined by Shapiro–Wilk test for normality.

^1^ LEA risk identified by ≥2.3 EDE-Q score and ≥8 LEAF-Q score for male and female participants respectively.

BMI: body mass index; IQR: interquartile range; LEA: low energy availability; SD: standard deviation.

**Table 2.** Differences based on LEA risk classification.

	At risk n = 12^1^	Not at risk n = 9	p value
Age (years)	33.17 ± 6.21	35 ± 8.80	0.581
Weight (kg)	77.17 ± 3.55	78.56 ± 9.23	0.78
Height (m)	1.76 ± 0.08	1.79 ± 0.07	0.412
BMI (kg/m^2^)	24.74 ± 1.96	24.47 ± 1.43	0.734
EDE-Q score^3^	9.28 ± 4.19	1.13 ± 0.91	<0.001
LEAF-Q score^2^	11.75 ± 1.71	4^4^	0.027

Data presented as mean ± SD unless specified otherwise.

^1^ LEA risk identified by ≥2.3 EDE-Q score and ≥8 LEAF-Q score for male and female participants, respectively.

^2^ Female participants only (n = 5).

^3^ Male participants only (n = 16).

^4^ Insufficient data to calculate standard deviation (total n = 5 female participants completed the LEAF-Q (n = 4 at risk, n = 1 not at risk)).

The p value obtained from independent sample t-test; significant difference between groups, p < 0.05.

BMI: body mass index; EDE-Q: Eating Disorder Evaluation Questionnaire; LEA: low energy availability; LEAF-Q: Low Energy Availability in Female Questionnaire; SD: standard deviation.

Differences in categorical variables between at risk and not at risk of LEA are summarised in Table 3. Due to limited regional and professional competitive participants, all competitive surfers were grouped together, and no significant differences were found. Raw data indicated that neither regional (n = 3) nor professional competitive (n = 1) surfers were classified as being at risk of LEA. Pearson's correlation identified a non-significant (p = 0.338) positive relationship between age and LEAF-Q of medium effect size (R = 0.549).

**Table 3.** Surfing ability, competitive status and daily activity by LEA risk classification.

	All participants n = 21	At risk^1^ n = 12	Not at risk n = 9	p value
Gender Male Female	16 (76) 5 (24)	8 (67) 4 (33)	8 (89) 1 (11)	0.237
Surfing ability				
Intermediate	9 (43)	6 (50)	3 (33)	
Advanced	12 (57)	6 (50)	6 (67)	0.445
Competitive status				
Not competitive	15 (71)	10 (83)	5 (56)	
Competitive	6 (29)	2 (17)	4 (44)	0.163
N exercise days/week				
1	0	0	0	
2	1 (5)	0	1 (11)	
3	0	0	0	
4	7 (33)	4 (33)	3 (33)	
5	2 (10)	1 (8)	1 (11)	
6	3 (14)	2 (8)	2 (22)	
7	8 (38)	6 (50)	2 (22)	0.539
Average time exercise/day				
0–1 h	7 (33)	4 (33)	3 (33)	
1–2 h	8 (38)	5 (42)	3 (33)	
3–6 h	5 (24)	2 (17)	3 (33)	
>6 h	1 (5)	1 (8)		0.695
Average time seated/day				
0–3 h	6 (29)	5 (42)	1 (11)	
3–6 h	11 (52)	5 (42)	6 (67)	
>6 h	4 (19)	2 (17)	2 (22)	0.305

Data presented as n(%)

^1^ LEA risk identified by ≥2.3 EDE-Q score and ≥8 LEAF-Q score for male and female participants, respectively.

The p value obtained from chi-square represents differences between LEA risk classifications; significant difference between groups, p < 0.05.

EDE-Q: Eating Disorder Evaluation Questionnaire; LEA: low energy availability; LEAF-Q: Low Energy Availability in Female Questionnaire.

Group differences between intermediate and advanced surfers are summarised in Table 4. Advanced surfers were significantly taller (p = 0.049) than intermediate surfers. No differences were identified between all other continuous variables namely EDE-Q and LEAF-Q scores. Group differences between competitive surfers and non-competitive surfers are summarised in Table 5 with no significant differences observed.

**Table 4.** Differences based on surfing ability in male and female surfers.

	Intermediate n = 9	Advanced n = 12	p value
Age (years)	30 (28 – 32)*	36.67 ± 8.28	0.093**
Weight (kg)	73.89 ± 12.77	80.76 ± 8.62	0.162
Height (m)	1.73 ± 0.08	1.80 ± 0.07	0.049
BMI (kg/m^2^)	24.28 ± 2.22	24.81 ± 1.29	0.620
EDE-Q^1^	4.83 ± 4.89	5.43 ± 5.52	0.832
LEAF-Q^2^	12 ± 2	7.5 ± 5	0.231

Data presented as mean ± SD unless specified otherwise.

*Value presented is median (IQR) due to data not being normally distributed as determined by Shapiro–Wilk test for normality.

**p value presented was calculated using a Mann–Whitney U test.

^1^ Female participants only (n = 5).

^2^ Male participants only (n = 16).

The p value obtained from independent sample t-test; significant difference between groups, p < 0.05.

BMI: body mass index; EDE-Q: Eating Disorder Evaluation Questionnaire; IQR: interquartile range; LEAF-Q: low energy availability in females questionnaire; SD: standard deviation.

**Table 5.** Differences between non-competitive and competitive surfers.

	Non-competitive n = 15	Competitive n = 6	p value	Effect size^3^
Age (years)	30 (28–40)*	35.17 ± 10.25	0.876	−0.23
Weight (kg)	76.3 ± 10.88	81.33 ± 10.89	0.354	−0.46
Height (m)	1.76 ± 0.08	1.80 ± 0.08	0.314	−0.5
BMI (kg/m^2^)	24.48 ± 1.78	24.99 ± 1.65	0.546	−0.297
EDE-Q^1^	5.15 ± 4.48	2.13 (0.88–12.13)*	1	−0.43
LEAF-Q^2^	12 ± 2	7.5 ± 4.95	0.116	1.367

Data presented as mean ± SD unless specified otherwise.

*Value presented is median (IQR) due to data not being normally distributed as determined by Shapiro–Wilk test for normality.

**p value presented was calculated using a Mann–Whitney U test.

^1^ Female participants only (n = 5).

^2^ Male participants only (n = 16).

^3^ Chohen D.

The p value obtained from independent sample t-test; significant difference between groups, p < 0.05.

BMI: body mass index; EDE-Q: Eating Disorder Evaluation Questionnaire; IQR: interquartile range; LEAF-Q: low energy availability in females questionnaire; SD: standard deviation.

Seventy unique 24-hour food logs were analysed and are summarised in Table 6. As it was not possible to link food records to individual participants, all participants were included in analysis, including n = 10 who did not meet the eligibility criteria. Mean energy intake was 2434.76 ± 1104.96 kcals/day. Only 23% of diet records met the recommended daily intake of carbohydrates for moderate exercise of 5 to 7g/kg body mass (Burke et al., 2011) and 57% exceeded the upper recommendation for saturated fat of <10% total energy (National Health and Medical Research Council, 2006). Only 44% of diet records met minimum calcium recommendations.

**Table 6.** Daily intake from 70 24-hour food logs of surfboard riders compared to recommendations.

Nutrient intake	24-Hour logs n = 70 (mean ± SD)	Recommendation	n (%) of 24-hour food logs above minimum recommendation*
Energy (kcal)	2434.76 ± 1104.96	-	-
Protein (g)	116.84 ± 55.11	-	-
Protein (g/kg/day)	1.56 ± 0.71	1.4–2^2**^	36 (51)
Fat (g)	95.41 ± 45.77	-	-
Fat (% total EI)	34.09 ± 16	20–35^1^	56 (80)
Saturated fat (g)	24.39 ± 19.06	-	-
Saturated fat (% total EI)	12.29 ± 6.81	10^1^	40 (57)
Carbohydrate (g)	261.30 ± 111.64	-	-
Carbohydrate (g/kg/day)	3.36 ± 1.44	5–7^3**^	16 (23)
Sugars (g)	103 ± 49.61	-	-
Dietary fibre (g)	32.88 ± 13.4	>25 (F), 30 (M)^1#^	52 (74)
Thiamin (mg)	1.66 ± 0.93	>1.1 (F), 1.2 (M)^1#^	47 (67)
Riboflavin	2.22 ± 1.21	>1.1 (F), 1.3 (M)^1#^	62 (89)
Naicin (mg)	55.12 ± 22.26	>14 (F), 16 (M)^1#^	70 (100)
Vitamin C (mg)	156.83 ± 122.54	>45^1^	58 (83)
Vitamin B6 (mg)	2.67 ± 3.13	>1.3 (F), 1.7 (M)^1#^	54 (77)
Vitamin B12 (mcg)	5.33 ± 3.96	>2.4^1^	56 (80)
Folate (mcg)	736.40 ± 351.08	>400^1^	59 (84)
Sodium (mg)	3144 ± 4446.41	>460–920^1^	69 (99)
Potassium (mg)	4342.72 ± 1725.88	>2800 (F), 3800 (M)^1#^	62 (89)
Magnesium (mg)	455.04 ± 172.12	>320 (F), 420 (M)^1#^	53 (76)
Calcium (mg)		>1000 (M), 1300 (F)^1#^	31 (44)
Phosphorus (mg)	1863.31 ± 799.25	>1000^1^	64 (91)
Iron (mg)	14.72 ± 6.18	>18 (F), 8 (M)^1#^	63 (90)
Zinc (mg)	12.45 ± 5.3	>8 (F), 14 (M)^1#^	55 (79)
Selenium (mcg)	92.13 ± 50.25	>60 (F), 70 (M)^1#^	50 (71)
Iodine (mcg)	181.45 ± 103.84	>150^1^	37 (53)

*Total 70 24-hour food logs collected and analysed.

**For nutrients expressed as a relative of body mass, mean body mass of all participants was used, inclusive of those who did not meet the eligibility criteria.

^#^For nutrients with male-specific and female-specific recommendations, the lowest value was used to assed adequacy.

^1^ National Health and Medical Research Council (2006); Nutrient reference values for Australia and New Zealand.

^2^ Jager et al. (2017) daily protein recommendations for building and maintaining muscle in most exercising individuals.

^3^ Burke et al. (2011) daily carbohydrate recommendations for moderate exercise (∼1hour/day).

EI: energy intake; F: adult female minimum recommended daily intake; M: adult male minimum recommended daily intake; SD: standard deviation.

## Discussion

This study primarily sought to identify LEA risk prevalence among surfers of intermediate ability and above. Of the 21 (male − 16, female − 5) eligible participants, 57% were deemed at risk.

The LEA risk prevalence of 80% found in females is markedly greater than previous studies featuring larger female samples of elite AFL players (Condo et al., 2019), elite soccer players (Luszczki et al. 2021), elite and recreational runners (Dervish et al., 2022; Sharps et al., 2022) and exercisers (Slater et al., 2016) of 30–53%. Whilst the shared use of the LEAF-Q is validated in female endurance athletes (Melin et al., 2014), findings by Magee et al. (2020) suggest that LEAF-Q does not overestimate LEA risk, but rather underestimates risk. They observed that direct EA assessment, indicated a 67% LEA risk prevalence, which was 10.7% above LEAF-Q. Despite direct EA assessment generally being considered cogent, caution is warranted due to notorious practical barriers and sources of error (Burke et al., 2018). These limitations may explain the discrepancy between LEA risk established by LEAF-Q and direct assessment by Magee et al. (2020). Given this, whilst indirect EA assessment is open to critique, the use of LEAF-Q is unlikely to explain the higher LEA risk prevalence we observed, and disparity with prior studies (Condo et al., 2019; Dervish et al., 2022; Luszczki et al. 2021; Sharps et al., 2022; Slater et al., 2016). As the present study was advertised on social media, it is also plausible that a sampling bias towards individuals with LEA symptoms may explain the higher prevalence in females we observed. A post hoc power analysis (power – 0.8) of the current study indicates a sample size of 23 female participants would be required for statistical significance, and future research should take heed when conceptualising research of similar nature.

A lack of validated male-specific LEA risk screening tools and LEA cut-offs (Sim and Burns 2021), limits validity, and likely contributes to the paucity of male EA research to date (Sundgot-Borgen et al., 2013). As the EDE-Q was designed to evaluate ED risk, not LEA risk, its use in the present study is a major limitation. For this reason, in male participants, LEA risk can be propounded only. Despite this, our observed male LEA risk of 50%, was markedly lower than a 70% risk prevalence using direct EA assessment observed in n = 20 elite male Gaelic football athletes (McGuire et al., 2022). Interestingly, EDE-Q was compared to direct EA assessment and only n = 4 (20%) were deemed at risk (McGuire et al., 2022). Like the LEAF-Q, this suggests that the EDE-Q underestimates LEA risk, which is not surprising as LEA can occur with or without DE/ED (Sim and Burns, 2021) and, as mentioned, the EDE-Q was synthesised to assess DE/ED risk, not LEA. However, it seems there is a close similarity between the total portion of female athletes and recreational exercises at risk of DE/ED compared to LEA. For instance, Dervish et al. (2022) found 49.4% and 47.3% risk prevalence of ED + DE, and LEA, respectively, supporting the notion that EDE-Q may indeed reliably predict LEA risk as previously proposed by Sim and Burns (2021).

One reason for the heterogeneity between the present study and prior EA investigations may be due to sport ability and/or competitive level. We found no significant association between surfing ability, and competitive status to both EDE-Q and LEAF-Q scores, suggesting that neither affect LEA risk in surfers. Critically, this finding may be misleading, as all competitive surfers were pooled together for statistical analysis as only three regional (female – 2, male – 1), and one (male) professional competitor participated. Of those n = 4, none were deemed at risk, including the single regional level female participant, suggesting that EA risk may be inversely associated with competitive level. Indeed, this is corroborated by Condo et al. (2019) who observed a relatively low LEA risk prevalence of 30% in a cohort of n = 30 elite level female AFL players. In contrast, competitive level has been positively associated with LEAF-Q score in female athletes and runners (Dervish et al., 2022; Sharps et al., 2022), and LEA risk is 1.7 to 1.8 times more likely in national and international level athletes compared to those who are recreationally active (Logue et al., 2019). The miniscule number of elite competition level surfers in our study may explain the discrepancy.

No significant correlations for body mass index and LEA risk were observed; however, a medium non-significant positive effect was found for age and LEAF-Q score (R = 0.549, p = 0.338) an observation consistent with prior study's (Dervish et al., 2022; Sharps et al., 2022). Therefore, it is plausible that age may predict LEA risk. The small sample size may explain the lack of a significant relationship observed.

We also found no significant associations for number of exercise days per week, hours of exercise, and hours spent seated, per day, for LEA risk. This is in contrast with Slater et al. (2016) who found that for every additional hour of exercise, LEA risk increased 1.13 times. Reasoning behind disparity is not clear, however, speculatively, the lack of significance may be explained by the use of broad categories to differentiate hours of exercises and time seated, instead of continuous scales.

Given the aforementioned limitations of the LEA risk screening tools used, dietary inadequacy indicated by dietary records supports the critical presence of LEA we observed. After all, LEA occurs when energy intake is insufficient relative to energy requirements (Mountjoy et al., 2018). We observed that only 23% of food records analysed indicated a daily carbohydrate intake beyond recommendations of 5 to 7 g/kg for moderate exercise of ∼1 hour/day (Burke et al., 2011). Considering that 38% and 24% of participants reported exercise of 1 to 2 and 3 to 6hours/day, respectively, carbohydrate recommendations used can be deemed conservative.

To bolster this, a pattern of insufficient carbohydrate intake and a high prevalence of LEA risk in intermittent high intensity sports are common. In elite male Gaelic football athletes (of which 70% at risk of LEA) mean daily carbohydrate intake was only 3.2 to 3.8 g/kg (McGuire et al., 2022), which is not dissimilar to the present study (3.36 ± 1.44g/kg). Furthermore, 91% of elite female AFL athletes (Lohman et al., 2019), and 96.3% of elite female soccer athletes (Magee et al., 2020) fell below carbohydrate recommendations (LEA risk 30% and 76%, respectively). Cumulatively, the inadequate carbohydrate intake we observed coincides with LEA risk.

Contrary to carbohydrate intake, mean daily protein intake (1.56 g/kg) exceeded the lower value of the ideal intake range (1.4–2g/kg (Jager et al., 2017)). This is consistent with Condo et al. (2019) who reported a similar mean daily protein intake of 1.5g/kg in elite female AFL athletes. However, in contrast, only 51% of participants in the present study exceeded the lower protein intake threshold (compared to 77.8% in Condo et al., 2019) suggesting that mean protein intake was heavily skewed by a few isolated very high protein consuming participants. This was supported by differences in standard deviation for absolute protein intake in the present study and Condo et al. (2019) (± 55.11 and ± 32.1, respectively). Furthermore, findings by Lohman et al. (2019) suggested elite athletes may consume more protein than sub-elite athletes (77% and 68% exceeded minimum protein recommendations, respectively), suggesting the presence of recreational surfers in the present study may explain the discrepancy.

Whilst caution should be taken due to the estimation of energy requirements using data from aquatic sport athletes, the only prior study investigating dietary intake of surfers found all 10 elite female surfers failed to meet energy balance (Felder et al., 1998). Calcium adequacy was only 44% and 60% in the present study, and Felder et al. (1998), respectively. Together, diet adequacy among surfers may be poor and exacerbated by practical barriers and poor food hygiene in remote competition locations (Felder et al., 1998).

Excluding misreporting errors of self-reported food records (Burke et al., 2018), there are limitations of this study of three-fold which pertain to a lack of association between individual diet records and participants. Firstly, nutrient intake expressed relative to body mass was compared to the total sample mean body mass. Secondly, the lower recommendations for micronutrients with unique male and female recommended dietary intake (RDI) values, was used, leading to underreporting of inadequacy. Finally, due to the anonymous nature of the food records, dietary intake analysis included food records from participants who did not meet the eligibility criteria (n = 10).

The seminal research investigated LEA risk in the surfing population and concluded an alarming portion (57%) of surfers are at risk of LEA and fail to consume adequate carbohydrates (77%), suggesting there is a need for surfing bodies, organisations, coaching staff and nutrition professionals to intervene, in order to attenuate EA-related health, performance and well-being risk factors. However, findings should be interpreted with caution due to methodological limitations inclusive of a small sample size, limited elite level competitors, the use of an ED risk assessment tool in male participants, the lack of validated male LEA screening tool, and direct EA assessment. Future investigations should (1) seek to recruit larger samples with a focus on females and higher-level competitors, (2) food intake records should be linked to individual participants for a more robust assessment of adequacy, (3) move past practical barriers and directly assess EA and (4) establish validated male screening tools and LEA cut-offs.

## Supplemental Material

sj-png-1-nah-10.1177_02601060231204927 - Supplemental material for Surfboard riders are at risk of low energy availability – A pilot study Supplemental material, sj-png-1-nah-10.1177_02601060231204927 for Surfboard riders are at risk of low energy availability – A pilot study by Mackenzie Baker, Pamela Magee and Josh Williamson in Nutrition and Health
