# Supplementary figures and images for: Surfboard riders are at risk of low energy availability – A pilot study
Source: Nutr Health. 2023 Sep 29;31(2):605–14. doi: 10.1177/02601060231204927 (PMC12174611; doi:10.1177/02601060231204927)

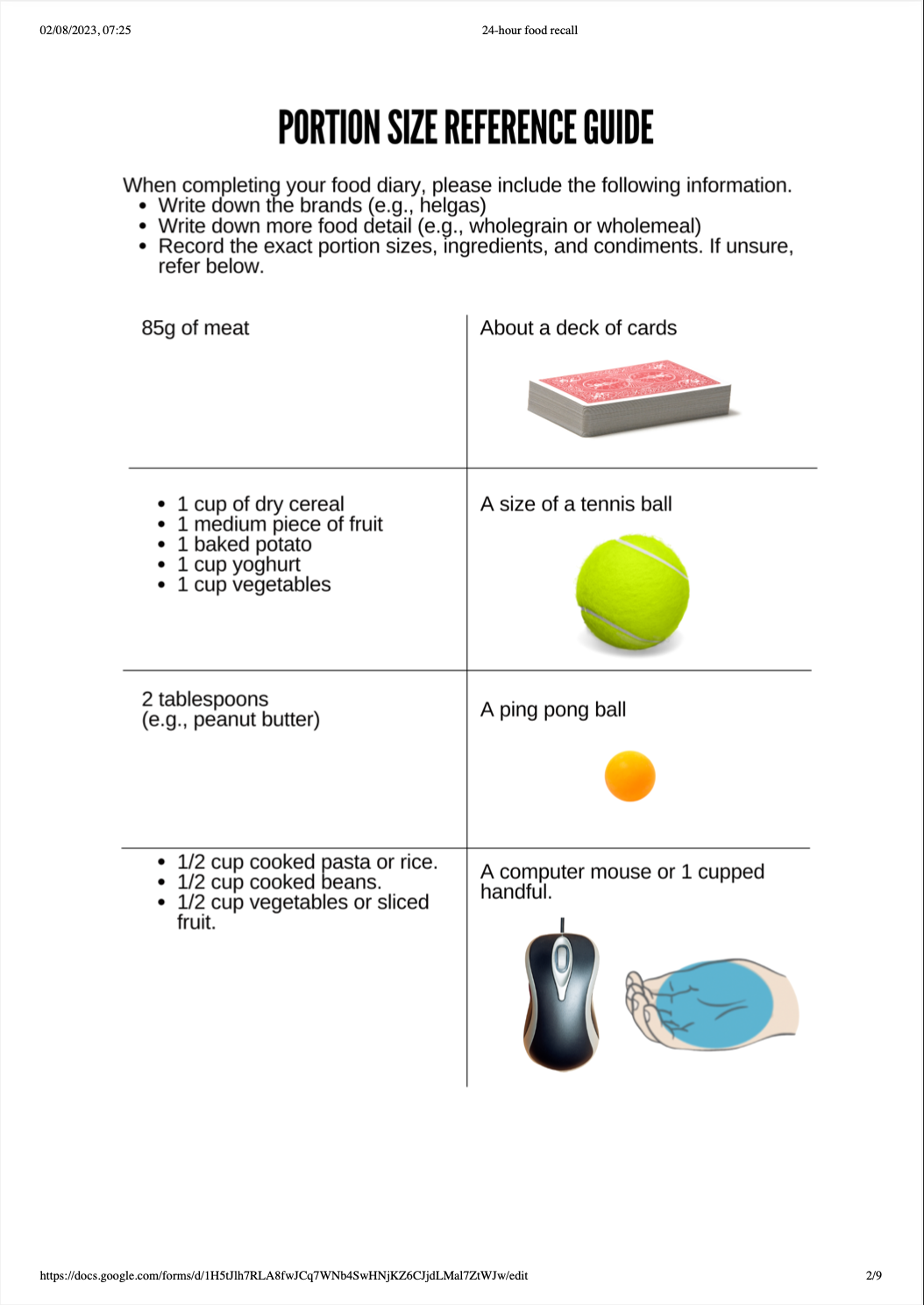

Supplement: sj-png-1-nah-10.1177_02601060231204927 - Supplemental material for Surfboard riders are at risk of low energy availability – A pilot study [file sj-png-1-nah-10.1177_02601060231204927.png]
